# High‐intensity focused ultrasound ablation combined with systemic therapy for unresectable colorectal cancer liver metastasis: A propensity score‐matched analysis

**DOI:** 10.1002/cam4.6774

**Published:** 2023-11-30

**Authors:** Fei Tang, Qin Zhong, Tingting Ni, Yingbo Xue, Jing Wu, Rong Deng, Qi Zhang, Yan Li, Xuanlu He, Zhenzhou Yang, Yu Zhang

**Affiliations:** ^1^ Department of Cancer Center, Second Affiliated Hospital Chongqing Medical University Chongqing China; ^2^ Department of Medical Oncology Guizhou Province People's Hospital Guiyang China; ^3^ National Health Commission Key Laboratory of Pulmonary Immune‐Related Diseases Guizhou Province People's Hospital Guiyang China

**Keywords:** ablation, colorectal cancer, high‐intensity focused ultrasound, metastatic hepatic carcinoma, prognosis

## Abstract

**Objective:**

Unresectable colorectal cancer liver metastasis (CRLM) remains a challenging obstacle that often prevents curative treatment. In this study, we retrospectively analyzed the efficacy and safety of high‐intensity focused ultrasound (HIFU) as a local adjuvant therapy for systemic chemotherapy for patients with unresectable CRLM. HIFU is a noninvasive method previously demonstrated as efficacious for various solid malignancies.

**Methods:**

Propensity score matching was used for the combination therapy group (HIFU group, *n* = 59) and the observation group receiving systemic therapy only (No‐HIFU group, *n* = 59). In addition, the survival benefit, adverse effects, and factors affecting prognosis following HIFU were evaluated.

**Results:**

The disease control rate was 77.9% and 62.7%, and the objective remission rate was 18.9% and 6.8% in the HIFU and non‐HIFU groups, respectively. The survival analysis showed that median progression‐free survival (mPFS) was 12.0 months and 11.0 months for the HIFU and non‐HIFU groups, respectively (*p* = 0.002). The univariate and multivariate analysis showed that pre‐treatment colorectal cancer liver metastasis lesion size was significantly associated with mPFS. In addition, patients that received a combination treatment for CRLM lesions <5.0 cm had a longer mPFS when compared to those receiving systemic therapy alone (13.0 months vs. 11.0 months, *p* = 0.001). In the HIFU group, patients with lesions <5.0 cm had a longer mPFS than patients with lesions ≥5.0 cm (13.0 months vs. 10.0 months, *p* = 0.04) (Figure 3B,C). Most treatment‐related adverse events observed in both groups were grade 1–2. Only four cases (6.8%) of grade 1–2 skin burns were observed in patients in the HIFU group; no other statistically significant adverse events were observed.

**Conclusions:**

Our study showed that HIFU ablation targeting unresectable CRLM alongside systemic therapy safely and significantly improved local control rates and prolonged mPFS, especially for lesions smaller than 5.0 cm.

## 1 INTRODUCTION

The liver is the most common site for colorectal cancer hematogenous metastasis, which is the leading cause of death for colorectal cancer (CRC) patients.^1^, ^2^ Patients with untreated liver metastases were reported to have a median survival time of only 6.9 months, with a 5‐year survival rate of less than 5% for unresectable patients.^3^, ^4^ Surgical resection is currently the best curative method for colorectal cancer liver metastases (CRLM). However, only 10%–20% of liver metastases can be initially resected due to multifocal disease, anatomical limitations, insufficient liver functional reserve, treatment‐related complications, and the patient's overall health conditions. Thus, unresectable liver metastases remain among the critical challenges when treating CRC patients with advanced disease.^3^, ^5^ Currently, a comprehensive treatment strategy is recommended for patients with unresectable CRLM, either at first diagnosis or after conversion therapy, including chemotherapy, molecular targeted therapy, immunotherapy, and local treatment for liver lesions such as radiofrequency ablation (RFA), anhydrous alcohol injection, and microwave ablation.^6^, ^7^ In addition, adjuvant local destructive therapy is currently considered an important tool for enhancing patient survival rates.^8^

High‐intensity focused ultrasound (HIFU) is a non‐invasive treatment that delivers external concentrated ultrasound energy to target lesions, increasing their temperature, which can cause irreversible coagulative necrosis. This process directly ablates solid tumors without damaging the intervening tissue.^9^, ^10^ The ablation procedure has been attempted to treat various malignancies, including primary and metastatic liver cancer, with promising results. However, most studies reporting HIFU‐based liver metastases from colorectal cancer management are single‐arm.^11^, ^12^

Our center has more than 10 years of experience using HIFU to treat a variety of solid tumors. In this retrospective study, we analyzed the efficacy and safety of adding HIFU therapy as local adjuvant therapy to systemic chemotherapy in patients with unresectable CRLM using a propensity score‐matched (PSM) analysis.

## 2 MATERIALS AND METHODS

### 2.1 Patients' characteristics

Colorectal cancer liver metastasis patients assessed as unsuitable for surgery in the Guizhou Provincial People's Hospital (CN) from January 2016 to December 2021 were included in this retrospective analysis. This assessment comprises patients classified as unresectable by the multidistrict team (MDT) and those not eligible for surgery due to their physical condition. Patients receiving HIFU treatment combined with chemotherapy ± targeted therapy were assigned to the combined treatment group (HIFU group). Patients receiving only chemotherapy ± targeted therapy were assigned to the observation group (No‐HIFU group). The inclusion criteria were as follows: (1) an age of ≥18 years; (2) no evidence of extrahepatic metastasis; (3) an Eastern Cooperative Oncology Group (ECOG) performance status of 0–2; (4) sufficient organ function (neutrophil count ≥1.5 × 109/L; hemoglobin ≥8 g/dL; platelets ≥75 × 109/L; alanine aminotransferase (ALT) or aspartate aminotransferase (AST) ≤2 × ULN, bilirubin ≤1.5 × ULN, creatinine ≤2 × upper limit of normal (ULN), creatinine clearance ≥60 mL/min/1.73 m^2^); (5) Child‐Pugh Class A or B. The exclusion criteria were as follows: (1) received prior thermal ablation (e.g., radiofrequency ablation or microwave therapy) or cryoablation treatment; (2) an ECOG performance status ≥3; (3) the lesion is outside the focusing range; (4) intestine is located on the ultrasound field center line or noticeable scars along the acoustic path; (5) intercostal stenosis or rib deformity. The clinicopathological data and treatment details were also collected. The Guizhou Province People's Hospital Institutional Review Board Committee approved the study protocol.

### 2.2 Treatment

All patients received a two‐drug, 5‐fluorouracil/leucovorin (or capecitabine) and oxaliplatin and/or irinotecan chemotherapy regimen. Additionally, some patients received Cetuximab monoclonal antibodies (KRAS, NRAS, BRAF gene wild‐type patients) or bevacizumab as an adjuvant targeted therapy. HIFU therapeutic procedure was administered simultaneously with the start of the first chemotherapy cycle.

High‐intensity focused ultrasound uses a high‐intensity focused tumor treatment system (model HIFU‐2001, Shanghai Jiao Tong University's Xindi Industrial Company, CN) with a real‐time ultrasound guidance device. The therapeutic ultrasonic working frequency was 50 Hz, with a 1 kW output power. The effective treatment depth ranged between 30 and 150 mm, with a focal volume of 3 mm × 3 mm × 8 mm and an effect focus of 6 mm × 6 mm × 10 mm. The HIFU therapeutic parameters were modified for each patient according to the tumor location and depth, the tumor tissue density, and the sound attenuation rate. Point accumulation was employed to create the treatment array, and large‐volume lesions were treated with fractional radiation.

The patient was prohibited from drinking water on the morning of treatment. During treatment, the patient was positioned prone on the HIFU treatment table, and the skin above the lesion's surface was brought into contact with degassed water. The tumor's location, size, and morphological characteristics were determined by diagnostic ultrasound, enhanced computed tomography (CT), or magnetic resonance imaging (MRI) before treatment. Then, the therapy area was repositioned using the HIFU system detection head. The treatment basin displacement covers the treatment area through points, lines, and surfaces. The entire HIFU treatment process is automated under the supervision of 1–2 physicians who monitor the patient's oxygen saturation, breathing, heart rate, and blood pressure throughout the treatment.

### 2.3 Evaluation of therapeutic efficacy and survival

The patients were evaluated for liver lesions by enhanced CT or MRI every 2–3 months after treatment. Tumor response was assessed using the modified Response Evaluation Criteria in Solid Tumors (mRECIST): Complete response (CR) was defined as the absence of enhancement for all target lesions during the arterial enhancement phase; partial response (PR) and progressive disease (PD) were defined as at least a 30% reduction or 20% increase (respectively) in the sum of the diameters of the enhancing lesions during the arterial phase, and neither PR nor PD was classified as stable disease (SD).^13^ The disease control rate (DCR) was defined as the proportion of patients with tumor shrinkage or stability and included those with CR, PR, and SD. The objective remission rate (ORR) was defined as the proportion of patients with CR or PR confirmed by follow‐up scans. Progression‐free survival (PFS) was calculated from the treatment date until the first imaging evidence of local tumor progression or the last follow‐up point. The follow‐up for this study ends in June 2022. Adverse events (AE) such as pain, fatigue, fever, and skin reactions were also analyzed using the National Cancer Institute Common Terminology Criteria for Adverse Events (CTCAE), version 5.0.^14^

### 2.4 Statistics

The data were analyzed using SPSS 25.0 statistical software. Unless otherwise stated, all the data were described using the mean ± standard deviation (SD) (for normally distributed data) or range median (for non‐normally distributed data). We used multivariate logistic regression to generate PSM for all eligible patients to balance the clinical variables between patients treated with or without HIFU. The primary disease, age, gender, TNM stage at initial diagnosis, treatment phase, ECOG performance status, number and size of liver metastases, body mass index (BMI), RAS (rat sarcoma viral oncogene homolog) status, and previous targeted therapy were entered into the model for matching. A 1:1 PSM analysis was computed without replacement using the nearest‐neighbor method with a stringent caliper of 0.01. The PFS analysis was performed using the Kaplan–Meier method, and the log‐rank test was used to compare prognostic factors. The univariate and multivariate analysis (Cox proportional risk model) assessed potential independent risk factors for PFS. Unless otherwise specified, the threshold for statistical significance was set as a two‐tailed *p* value of less than 0.05 (*p* < 0.05).

## 3 RESULTS

### 3.1 Clinical characteristics

A total of 67 patients were treated with HIFU, and 205 were without HIFU based on the above inclusion and exclusion criteria (Table S1). A 1‐to‐1 matching was performed with a caliper width of 0.2 standard deviations, and after propensity score matching, 118 patients were included in the analysis, 59 in HIFU group and 59 in non‐HIFU group, with balanced baseline characteristics in both cohorts. The clinicopathological information for these patients is shown in Table 1. Patients with multi‐line treatment and multiple liver metastases formed the majority for both groups.

**TABLE 1:** Clinical characteristics for the CRLM patients prior to high‐intensity focused ultrasound (HIFU) treatment.

Characteristics	HIFU group (*N* = 59)	Non‐HIFU group (*N* = 59)	*p* Value
Primary Disease, *n* (%)			
Colon	30 (50.8)	26 (44.1)	0.461
Rectum	29 (49.2)	33 (55.9)	
Age (years), mean ± SD	58.5 ± 11.7	58.9 ± 12.3	
Median age (years), range	59 (29–79)	57 (35–86)	0.854
>mean, *n* (%)	29 (49.2)	28 (47.5)	
≤mean, *n* (%)	30 (50.8)	31 (52.5)	
Gender, *n* (%)			
Male	32 (54.2)	33 (55.9)	0.853
Female	27 (45.8)	26 (44.1)	
Stage at initial diagnosis, *n* (%)			
II–III	22 (37.3)	20 (33.9)	0.406
IV	37 (62.7)	39 (66.1)	
Treatment phase, *n* (%)			
First‐line therapy	16 (27.1)	12 (20.3)	0.387
Multi‐line therapy	43 (72.9)	47 (79.7)	
ECOG performance status, *n* (%)			
0–1	53 (89.8)	50 (84.7)	0.407
2	6 (10.2)	9 (15.3)	
Number of liver metastasis lesions, *n* (%)			
Single	14 (23.7)	17 (28.8)	0.530
Multiple	45 (73.6)	42 (71.2)	
Liver metastasis lesion size (cm), mean ± SD	5.0 ± 1.4	4.9 ± 1.1	
Median lesion size (cm), range	4.8 (2.4–8.9)	4.7 (2.5–7.3)	
>mean, *n* (%)	28 (47.5)	29 (49.2)	0.854
≤mean, *n* (%)	31 (52.5)	30 (50.8)	
BMI, mean ± SD	24.3 ± 2.8	24.2 ± 2.7	
>mean, *n* (%)	28 (47.5)	31 (52.5)	0.581
≤mean, *n* (%)	31 (52.5)	28 (47.5)	
RAS status, *n* (%)			
Mutant	23 (38.9)	21 (35.6)	0.703
Wild‐type	36 (61.1)	38 (64.4)	
Targeted therapy, *n* (%)			
With	45 (73.6)	42 (71.2)	0.530
Without	14 (23.7)	17 (28.8)	

Abbreviations: BMI, body mass index; CRLM, colorectal liver metastases; ECOG, Eastern Cooperative Oncology Group; RAS, rat sarcoma viral oncogene homolog.

### 3.2 Evaluation of HIFU treatment

**FIGURE 1:**
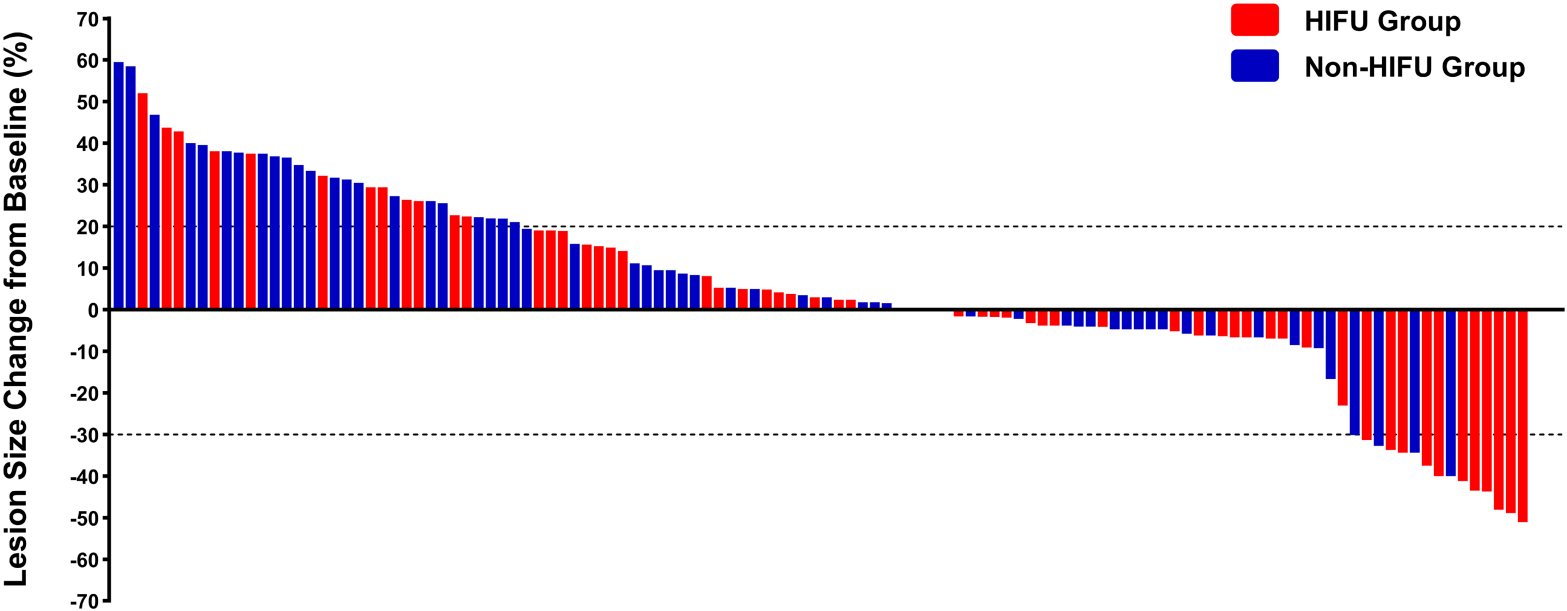
Liver metastasis response evaluation after treatment.

**FIGURE 2:**
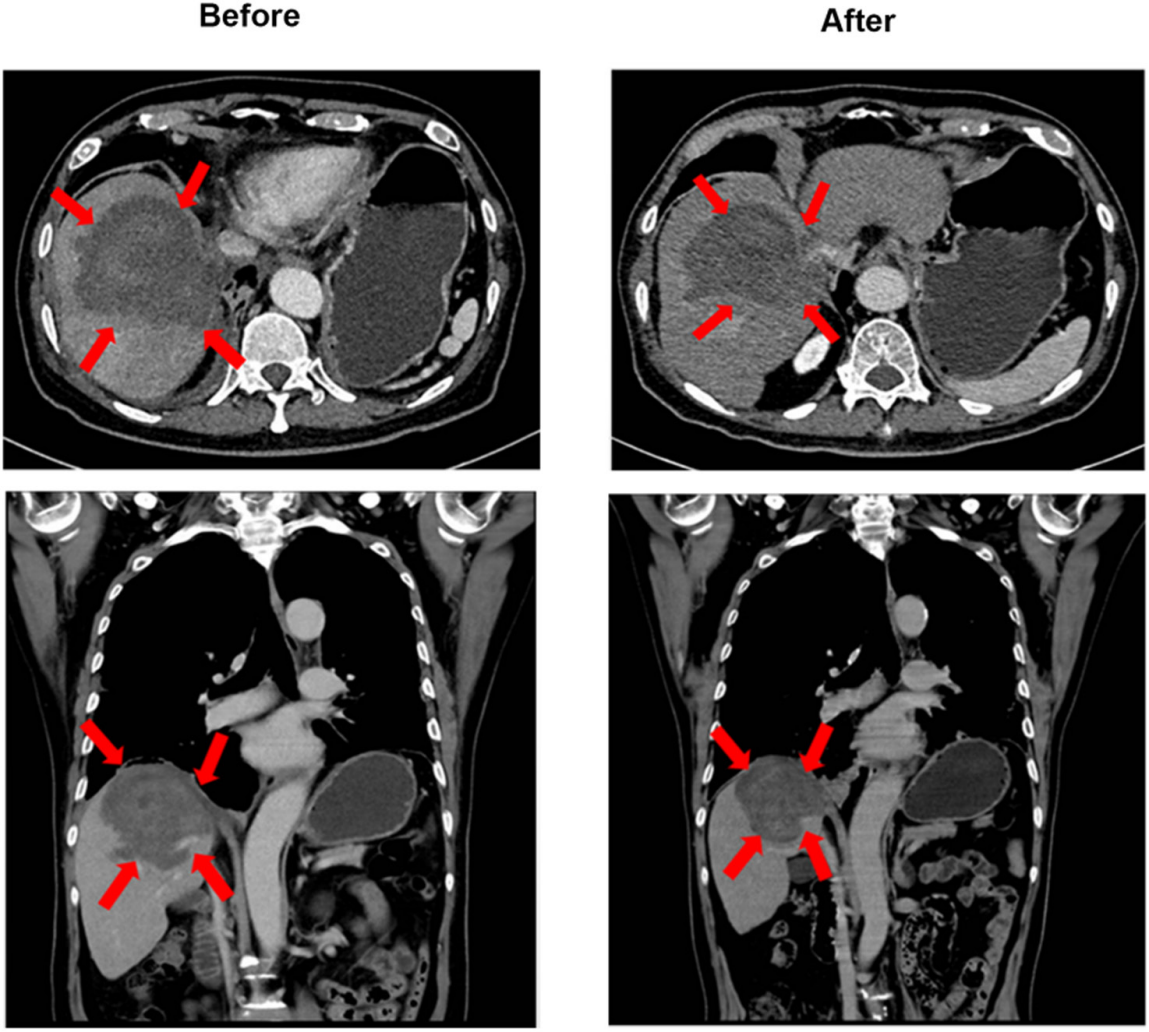
Representative images demonstrating liver metastasis lesion reduction following HIFU combined with systemic chemotherapy treatment.

Each patient in the HIFU group received an average of 9.64 ± 0.86 (range: 5–10) HIFU ablations during treatment. The treatment intensity and average treatment power are shown in Table 2. No CR was observed in either group, while more patients in the HIFU group achieved PR (18.6% vs. 6.8%), and the majority of patients in both groups were SD. 77.9% (95% confidence interval [CI], 67.1–88.9) and 18.9% (95% CI, 8.4–28.9) of patients in the HIFU group had DCR and ORR, respectively, compared with 62.7% (95% CI, 50.0–75.4) and 6.8 (95% CI, 0.2–13.4) in the non‐HIFU group. Figure 1 depicts the changes in the index tumor size from baseline. Representative images displaying the lesions before and after HIFU (combined with systemic chemotherapy) treatment are shown in Figure 2.

**TABLE 2:** High‐intensity focused ultrasound (HIFU) ablation parameters and treatment response.

Parameters and Response	HIFU group (*N* = 59)	Non‐HIFU group (*N* = 59)
Number of fractions within one course, *N* ± SD (range)	9.84 ± 0.54 (7–10)	–
Treatment duration (minutes), mean ± SD (range)	26.58 ± 1.40 (23–30)	–
Sonication time (seconds), mean ± SD (range)	476.45 ± 45.45 (420–600)	–
Treatment intensity (seconds /hours), mean ± SD (range)	1078.98 ± 118.85 (868.94–1350)	–
Average power (watts), mean ± SD (range)	673.14 ± 70.65 (500–800)	–
CR, *n* (%)	0	0
PR, *n* (%)	11 (18.6)	4 (6.8)
SD, *n* (%)	35 (59.3)	33 (55.9)
PD, *n* (%)	13 (22.0)	22 (37.3)
DCR (%) [95% CI]	77.9 [67.1–88.9]	62.7 [50.0–75.4]
ORR (%) [95% CI]	18.9 [8.4–28.9]	6.8 [0.2–13.4]
mPFS (months)	12.2 [10.3–13.7]	11.0 [10.3–11.7]

Abbreviations: CR, complete response; DCR, disease control rate; mPFS, median progression‐free survival; ORR, objective remission rate; PD, progressive disease; PR, partial response; SD, stable disease.

### 3.3 Survival analysis

**FIGURE 3:**
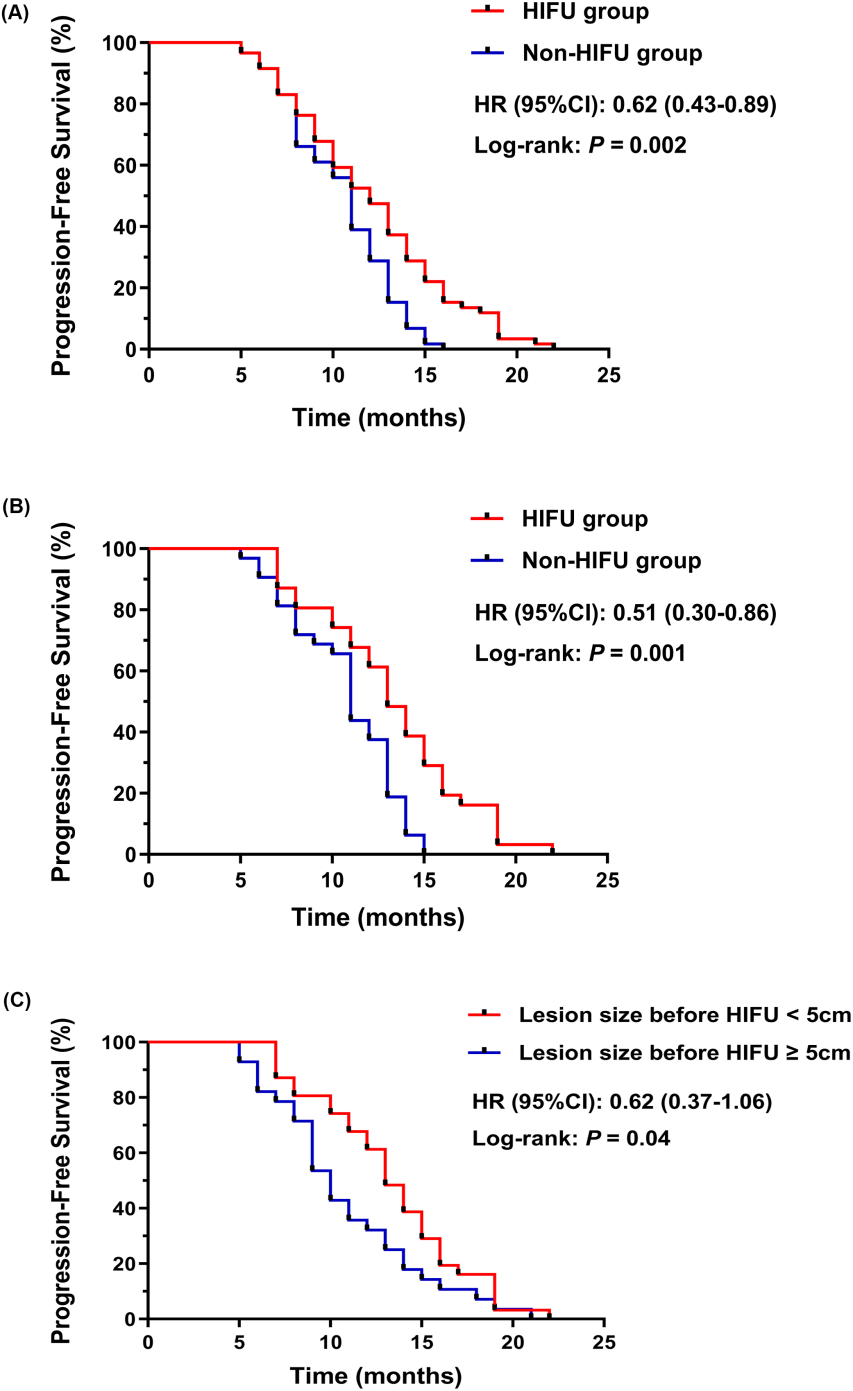
Progression‐free survival for patients with colorectal cancer liver metastasis with different treatment strategies and lesion characteristics.

The survival analysis in this study showed that the median PFS (mPFS) was 12.0 months [95% CI: 10.3–13.7] and 11.0 months [95% CI: 10.3–11.7] (*p* = 0.002) in the HIFU and non‐HIFU groups, respectively (Figure 3A). Both univariate and multivariate analysis showed that the CRLM lesion size before treatment was significantly associated with mPFS. A 5.75‐cm lesion size cut‐off value was obtained by plotting the ROC curve (Table 3). A stratified analysis showed that patients with pre‐treatment liver metastases lesions <5.0 cm had a longer mPFS when receiving HIFU combined with systemic therapy compared with systemic therapy alone (13.0 months [95% CI: 11.4–14.6] vs. 11.0 months [95% CI: 10.2–11.8]; *p* = 0.001), while in the HIFU group, patients with lesions <5.0 cm had a longer mPFS than those with lesions ≥5.0 cm (13.0 months [95% CI: 11.4–14.6] vs. 10.0 months [95% CI: 8.7–11.3]; *p* = 0.04) (Figure 3B,C).

**TABLE 3:** Prognostic univariate and multivariate cox analysis in the matched data set.

	Univariate analysis		Multivariate analysis	
	Hazard ratio (95% CI)	*p* Value	Hazard ratio (95% CI)	*p* Value
Primary disease				
Rectum (reference)	1		1	
Colon	0.82 (0.54–2.53)	0.49	0.79 (0.39–1.58)	0.50
Age				
<50 (reference)	1		1	
50–60	0.87 (0.38–2.02)	0.75	1.00 (0.38–2.63)	0.99
61–70	1.00 (0.45–2.18)	0.99	1.31 (0.52–3.28)	0.56
>70	1.34 (0.62–2.91)	0.46	1.90 (0.77–4.67)	0.16
Gender				
Male (reference)	1		1	
Female	1.12 (0.67–1.89)	0.66	1.12 (0.62–2.01)	0.72
Stage at initial diagnosis				
IV (reference)	1		1	
II–III	0.84 (0.49–1.44)	0.52	1.27 (0.65–2.50)	0.49
Treatment phase				
Multi‐line therapy (reference)	1		1	
First‐line therapy	0.58 (0.32–1.05)	0.06	1.62 (0.77–3.41)	0.20
ECOG performance status				
0–1 (reference)	1		1	
2	0.68 (0.29–1.58)	0.36	1.42 (0.56–3.59)	0.46
Number of liver metastasis lesions				
Single (reference)	1		1	
Multiple	1.19 (0.64–2.22)	0.58	0.73 (0.34–1.57)	0.42
Liver metastasis lesion size				
Lesions size <5.75 cm (reference)	1		1	
Lesions size ≥5.75 cm	0.57 (0.32–0.99)	0.04*	2.24 (1.10–4.56)	0.03*
RAS status				
Mutant (reference)	1		1	
Wild‐type	1.07 (0.82–1.40)	0.62	1.00 (0.54–1.83)	0.99
Targeted therapy				
With (reference)	1		1	
Without	1.22 (0.66–2.25)	0.53	0.99 (0.50–1.95)	0.97

Abbreviations: ECOG, Eastern Cooperative Oncology Group; RAS, rat sarcoma viral oncogene homolog.

*p < 0.05.

### 3.4 Safety

Most of the treatment‐related adverse events observed in both groups were grade 1–2, with neutrophil count decreased, fatigue, hepatic insufficiency, and fever accounting for the main adverse effects, and only four cases (6.8%) of grade 1–2 skin burns were observed in patients in the HIFU group, with no statistical difference in the remaining adverse events. The patients had no treatment‐related grade 5 adverse events (Table 4).

**TABLE 4:** Adverse events experienced by CRLM patients after high‐intensity focused ultrasound (HIFU) treatment.

Adverse event	HIFU group (*N* = 59)			Non‐HIFU group (*N* = 59)			*p* Value
	Grades 1–2 *n*, (%)	Grades 3–4 *n*, (%)	Grade 5 *n*, (%)	Grades 1–2 *n*, (%)	Grades 3–4 *n*, (%)	Grade 5 *n*, (%)	
Skin burns	4 (6.8)	0	0	0	0	0	
Fever	6 (10.2)	0	0	4 (6.8)	0	0	0.416
Fatigue	11 (18.6)	3 (5.1)	0	10 (16.9)	3 (5.1)	0	0.918
Abdominal distension	4 (6.8)	0	0	3 (5.1)	0	0	0.285
Abdominal pain	5 (8.5)	1 (1.7)	0	3 (5.1)	1 (1.7)	0	0.747
Diarrhea	4 (6.8)	0	0	5 (8.5)	0	0	0.853
Platelet count decreased	3 (5.1)	1 (1.7)	0	3 (5.1)	0	0	0.350
Neutrophil count decreased	9 (15.3)	2 (3.4)	0	8 (13.6)	4 (6.8)	0	0.408
Alanine aminotransferase increased	6 (10.2)	3 (5.1)	0	6 (10.2)	2 (3.4)	0	0.707
Aspartate aminotransferase increased	6 (10.2)	3 (5.1)	0	5 (8.5)	2 (3.4)	0	0.838
INR increased	2 (3.4)	0	0	2 (3.4)	0	0	

Abbreviations: CRLM, colorectal liver metastases; INR: international normalized ratio.

## 4 DISCUSSION

The current treatment methods for unresectable CRLM are based on systemic therapy, including chemotherapy ± targeted therapy or immune checkpoint inhibitor therapy indicated for patients with microsatellite instability‐high (MSI‐H) or mismatch repair‐deficient (dMMR).^15^, ^16^, ^17^, ^18^, ^19^ Moreover, guidelines for unresectable CRLM allow for selecting appropriate local destructive treatment measures alongside systemic chemotherapy to enhance the control of local lesions according to their location, treatment goals, treatment‐related complications, and the patient's condition.^20^ Therefore, security and effectiveness evaluation of combination therapy strategies is necessary.

Previous studies have reported the exploration and shown positive clinical outcomes of various local ablation techniques in CRLM, such as RFA, microwave, and cryotherapy ablation.^21^, ^22^ The most applied is RFA, and in a phase II study, the mPFS for RFA combined with systemic therapy versus systemic therapy alone was 16.8 months (95% CI, 11.7–22.1) and 9.9 months (95% CI, 9.3–13.7), respectively.^23^ However, for technical reasons, RFA is currently more recommended for lesions <3 cm because the failure rate is significantly higher for larger lesions.^24^, ^25^ In comparison, the mean diameter for lesions in the HIFU group in this study reached 4.8 cm. A subgroup analysis showed that higher mPFS was achieved for lesions <5.0 cm compared to the non‐HIFU group, suggesting that HIFU may provide more treatment options for patients with larger lesions. It is worth emphasizing that unintentional collateral damage generally occurs at a higher rate for RFA. Percutaneous approach is currently the most common procedure for RFA, and this invasiveness may lead to multiple complications such as intra‐abdominal hemorrhage, tract seeding, liver abscess, and intestinal perforation.^8^ It has been reported that patients with liver dysfunction and thrombocytopenia often exhibit intolerance to RFA because the percutaneous puncture needle may cause bleeding in patients with coagulation disorders and low platelet counts due to vascular‐rich tumors, and these two comorbidities are common in patients with CRLM receiving systemic therapy.^26^, ^27^ In contrast, the non‐invasive property of HIFU ablation well avoids the risks mentioned above, and since no probe needs to be implanted into the vivo, the technique circumvents the possibility of bleeding, infection, and tumor implantation and is equally suitable for larger lesions.^28^, ^29^ Microwave ablation requiring percutaneous puncture also suffers from these problems, and similar complication rates have been reported for patients with liver metastases treated with microwave ablation and RFA.^27^, ^30^, ^31^, ^32^ In addition, microwave ablation is relatively costly and less definitive visualization intra‐procedurally during active ablation,^33^ whereas HIFU ablation offers the advantages of lower treatment costs and monitoring during treatment. Most of the current studies of cryotherapy for the treatment of colorectal cancer liver metastases have been conducted in comparison with conventional surgery, and its efficacy in unresectable liver metastases is unclear, while cryotherapy has also been reported to have a higher risk of complications than radiofrequency ablation.^34^ HIFU ablation focuses ultrasound energy from outside the body on the target lesion in the body, causing coagulative necrosis and ablation of the target lesion without damaging adjacent organs and tissues. In our study, no intolerable adverse effects occurred in either group. Skin burns were unique to patients treated with HIFU compared to controls but were mild and resolved quickly without specific treatment. It is demonstrated that HIFU is a safe and feasible option for patients with unresectable CRLM, and studies comparing HIFU with other local destructive therapies in these patients may be required.

High‐intensity focused ultrasound ablation has demonstrated definite efficacy in treating a variety of solid malignancies as a noninvasive antitumor interventional therapy.^35^ In recent years, its therapeutic potential in CRLM has gradually gained attention.^11^ However, in previous reports, HIFU has often been evaluated as monotherapy, and the combination studies were all single‐arm.^36^, ^37^ In a recent retrospective study, 42 patients with CRLM received systemic therapy after HIFU ablation, which achieved a median overall survival of up to 31 months.^12^ Still, the efficacy could not be independently evaluated for HIFU alone because it was a single‐arm study. Therefore, in this study, we applied a PSM approach that minimizes selection bias and indication confounding to create two comparable groups similar to a randomized controlled trial to assess the efficacy of HIFU therapy as a local adjuvant treatment. In addition, this study treated all patients with HIFU concurrently with systemic therapy. We did not observe any significant increase in toxicities during the treatment period, indicating that receiving HIFU therapy does not have to interfere with systemic therapy. To our knowledge, this is the first cohort study of HIFU plus concurrent systemic chemotherapy for CRLM.

There are several limitations in the present study. Firstly, although this study used the statistical method of PSM to balance baseline characteristics, it was still a retrospective and inevitably biased study. In particular, this was an analysis based on real‐world data, and systemic treatment strategies vary depending on the patient's condition and the flexibility of physicians' clinical decisions. However, this limitation was present in both groups, so this study may still provide some clues to clinical practice. In addition, this was a single‐center study involving only Chinese patients; thus, its representation may be limited, and a large prospective multicenter randomized trial is expected.

## 5 CONCLUSIONS

Our study showed that HIFU ablation therapy targeting liver metastases and systemic therapy safely and significantly improved local control rates and prolonged mPFS in patients with unresectable CRLM, especially for smaller‐diameter lesions (<5.0 cm in this study).

## AUTHOR CONTRIBUTIONS

**Fei Tang:** Data curation (equal); formal analysis (equal); investigation (equal); writing – original draft (lead). **Qin Zhong:** Data curation (equal); formal analysis (equal); investigation (equal); writing – original draft (equal). **Tingting Ni:** Data curation (equal); formal analysis (equal); investigation (supporting); writing – original draft (supporting). **Yingbo Xue:** Software (equal); validation (lead). **Jing Wu:** Software (equal); validation (equal). **Rong Deng:** Resources (lead). **Qi Zhang:** Visualization (lead). **Yan Li:** Visualization (equal). **Xuanlu He:** Visualization (supporting). **Zhenzhou Yang:** Project administration (lead). **Yu Zhang:** Conceptualization (lead); methodology (lead); supervision (lead); writing – review and editing (lead).

## FUNDING INFORMATION

This project was supported by Science and Technology Fund Project of Guizhou Health Commission (gzwjkj 2020‐1‐032 and gzwkj 2022‐028); Lian Yun Gang Shi Hui Lan Public Foundation (HL‐HS2020‐33); Science and Technology Foundation of Guizhou Province general project (Qiankehejichu‐ZK [2023] General 212); Youth Foundation of Guizhou Provincial People's Hospital (GZSYQN [2021] 03 and GZSYQN [2022] 09); Doctor Foundation of Guizhou Provincial People's Hospital (GZSYBS [2022] 09).

## CONFLICT OF INTEREST STATEMENT

The authors declare that they have no conflict of interest.

## ETHICS STATEMENT

The Guizhou Province People's Hospital Institutional Review Board Committee approved the study protocol.

## CONSENT

All patients included in the study provided written informed consent. Written informed consent has been obtained from individuals for any potentially identifiable images or data in this publication.

## Supporting information


Table S1.

## Data Availability

The datasets used in this study are available from the corresponding author upon reasonable request.
