# Supplementary material for: High‐intensity focused ultrasound ablation combined with systemic therapy for unresectable colorectal cancer liver metastasis: A propensity score‐matched analysis
Source: Cancer Med. 2023 Nov 30;12(24):21985–95. doi: 10.1002/cam4.6774 (PMC10757091; doi:10.1002/cam4.6774)
Supplement: Supplementary file 1 — Table S1. [file CAM4-12-21985-s001.docx]

**Table S1. Baseline clinical characteristics of CRLM patients before propensity score matching**

| Characteristics | HIFU group (N = 67) | Non-HIFU group (N=205) | *P* value |
| --- | --- | --- | --- |
| Primary Disease, *n (%)* |  |  |  |
| Colon | 37 (55.2) | 94 (45.9) | 0.183 |
| Rectum | 30 (44.8) | 111 (54.1) |  |
| Age (years), mean ± SD | 58.3 ± 12.2 | 59.9 ± 11.9 |  |
| Median age (years), range | 60 (18-79) | 60 (27-89) |  |
| > mean, *n (%)* | 33 (49.3) | 72 (35.1) | 0.039 |
| ≤ mean, *n (%)* | 34 (50.7) | 133 (64.9) |  |
| Gender, *n (%)* |  |  |  |
| Male | 34 (50.7) | 108 (52.7) | 0.783 |
| Female | 33 (49.3) | 97 (47.3) |  |
| Stage at initial diagnosis, *n (%)* |  |  |  |
| Ⅱ-Ⅲ | 25 (37.3) | 85 (41.5) | 0.548 |
| Ⅳ | 42 (62.7) | 120 (58.5) |  |
| Treatment phase, *n (%)* |  |  |  |
| First-line therapy | 18 (26.9) | 46 (22.4) | 0.458 |
| Multi-line therapy | 49 (73.1) | 159 (77.6) |  |
| ECOG performance status, *n (%)* |  |  |  |
| 0-1 | 61 (91.0) | 177 (86.3) | 0.056 |
| 2 | 6 (9.0) | 28 (13.7) |  |
| Number of liver metastasis lesions, *n (%)* |  |  |  |
| Single | 14 (20.9) | 70 (34.1) | 0.042 |
| Multiple | 53 (79.1) | 135 (65.9) |  |
| Liver metastasis lesion size (cm), mean ± SD | 5.0 ± 1.4 | 5.2 ± 1.2 |  |
| Median lesion size (cm), range | 4.8 (2.4-8.9) | 5.2 (2.5-8.4) |  |
| > mean, *n (%)* | 30 (44.8) | 98 (47.8) | 0.666 |
| ≤ mean, *n (%)* | 37 (55.2) | 107 (52.2) |  |
| BMI, mean ± SD | 24.2 ± 2.8 | 24.4 ± 2.6 |  |
| > mean, *n (%)* | 33 (49.3) | 105 (51.2) | 0.780 |
| ≤ mean, *n (%)* | 34 (50.7) | 100 (48.8) |  |
| RAS status, *n (%)* |  |  |  |
| Mutant | 25 (37.3) | 79 (38.5) | 0.858 |
| Wild-type | 42 (62.7) | 126 (61.5) |  |
| Targeted Therapy, *n (%)* |  |  |  |
| With | 53 (79.1) | 155 (75.6) | 0.558 |
| Without | 14 (20.9) | 50 (24.4) |  |

CRLM: colorectal liver metastases, ECOG: Eastern Cooperative Oncology Group, BMI: body mass index, RAS: rat sarcoma viral oncogene homolog.
